# Executive functions in mono- and bilingual children with language impairment – issues for speech-language pathology

**DOI:** 10.3389/fpsyg.2015.01074

**Published:** 2015-07-27

**Authors:** Olof Sandgren, Ketty Holmström

**Affiliations:** Department of Logopedics, Phoniatrics, and Audiology, Clinical Sciences, Lund UniversityLund, Sweden

**Keywords:** bilingualism, language impairment, executive functions, bilingual advantage, speech-language pathology

## Abstract

The clinical assessment of language impairment (LI) in bilingual children imposes challenges for speech-language pathology services. Assessment tools standardized for monolingual populations increase the risk of misinterpreting bilingualism as LI. This Perspective article summarizes recent studies on the assessment of bilingual LI and presents new results on including non-linguistic measures of executive functions in the diagnostic assessment. Executive functions shows clinical utility as less subjected to language use and exposure than linguistic measures. A possible bilingual advantage, and consequences for speech-language pathology practices and future research are discussed.

## Executive Functions in Bilingual Children

The executive functions of bilingual children have repeatedly been shown to exceed those of monolingual peers. Bilingual children outperform monolinguals on measures of inhibition, task switching, and working memory. Bialystok (1999) used a dimensional change card sort task to assess bilingual 3- to 6-year-old children’s attentional control when the principle for sorting the cards changed from color to shape. The results revealed a bilingual advantage interpreted as a superior ability to inhibit incorrect responses. An ensuing experiment further traced the bilingual advantage to a specific superiority in disregarding no longer relevant information, most evident for perceptual, rather than semantic, features, and for tasks of greater complexity (Bialystok and Martin, 2004). A greater bilingual advantage in more complex tasks was also confirmed by Bialystok (2011) who showed greater performance in tasks with high demands on executive functions and on coordinating visual and auditory information. However, the bilingual advantage in attentional control extends beyond the visual domain. Using non-verbal and verbal go/no-go tasks, requiring participants to alternatingly respond to non-verbal sounds (e.g., a barking dog and a ringing bell) and verbal auditory stimuli (e.g., /pa/ and /ba/), Foy and Mann (2014) found a bilingual advantage regarding both accuracy and response times for non-verbal, but not verbal, trials.

A bilingual advantage has also been found for working memory, again with larger effects for complex tasks imposing greater executive function demands. Morales et al. (2013) hypothesized that bilingual children would exhibit better working memory as an effect of its central role in the executive functions necessary to control and coordinate two language systems. The authors contrasted congruent trials, with isolated working memory demands (remembering rules), and incongruent trials with additional demands on executive control (remembering rules and following shifting instructions while ignoring distraction). While bilingual and monolingual 5-year-old children performed similarly on the congruent trials, with minimal demands on executive functions beyond working memory, the bilingual children responded faster. On the incongruent trials, with greater overall executive function demands, the bilingual advantage was shown by both greater accuracy and faster responses (Morales et al., 2013). Similar results had previously been presented by Carlson and Meltzoff (2008) who found a bilingual advantage for conflict tasks, similar to the incongruent trials, but not for less complex delay tasks, only requiring working memory. Furthermore, the bilingual advantage in executive functions outweighed a socio-economic disadvantage and lower language scores in the bilingual group (Carlson and Meltzoff, 2008).

To summarize, the results point to domain-general beneficial effects of bilingualism on executive functions, as further confirmed by a meta-analysis of 63 studies on the cognitive outcomes of bilingualism (Adesope et al., 2010) revealing the largest mean effect sizes for attentional control (0.96), abstract and symbolic representation (0.57), and working memory (0.48). Furthermore, the bilingual advantage grows with increasing task complexity and increasing executive function demands.

## Executive Functions in Children with Language Impairment

In contrast to the advantage in executive functions evidenced by bilinguals with typical language development, monolingual children with LI have been found to be at a disadvantage compared to peers with typical language development. Im-Bolter et al. (2006) found 7- to 12-year-old children with LI to score lower than same-age peers on tasks requiring inhibition of responses and addition of information to be held in working memory. Vugs et al. (2014) found working memory deficits of 4- to 5-year-olds with LI to extend beyond the verbal domain to also include visuospatial working memory deficits, a finding taken as evidence of domain-general effects of LI with impact also on non-verbal aspects of cognition (for similar results, see Hoffman and Gillam, 2004). With 89 percent of the participants identified correctly as either LI or typically developing (TD), the authors could establish the clinical utility of working memory assessment in clinical decision making. Furthermore, using parent ratings of children’s executive functions, the authors were able to document deficits in several executive functions, including inhibition (Vugs et al., 2014).

Henry et al. (2012) examined the executive functions of children with diagnosed LI in comparison to peers with undiagnosed low language/cognitive functioning, and TD. The authors found lower executive functions for participants with LI, with particular deficits in areas including verbal and non-verbal working memory, and non-verbal inhibition. Furthermore, the group difference remained significant despite adjustment for verbal IQ, indicating that the findings could not be attributed to reduced language ability. Similarly to Vugs et al. (2014), the authors found support for a domain-general impairment, and pointed to the possible clinical meaningfulness of evaluating executive functions in the assessment of LI. Furthermore, the group with undiagnosed language problems performed similarly to the group with LI on almost all measures, further supporting the clinical utility of the measures (Henry et al., 2012).

The findings of negative domain-general consequences of LI have inspired research and implementation of non-linguistic cognitive treatments to remediate the effects. While showing improvements in trained areas, establishing that executive functions are modifiable by intervention (see, e.g., Thorell et al., 2009; Holmes and Gathercole, 2014) research has yet to provide conclusive evidence of transfer to other executive functions (see, e.g., Melby-Lervåg and Hulme, 2013) or effects exceeding those of targeted language intervention (Ebert et al., 2014). However, small scale studies using single-case experimental designs have shown promising results, indicating a causal rather than merely correlational association between non-linguistic processing and language ability, in need of replication in larger samples (see, Ebert and Kohnert, 2009; Ebert et al., 2012).

## Executive Functions in Bilingual Children with Language Impairment

The interaction of bilingualism and LI on executive functions remains largely unexplored. As indicated by the results above, bilingualism appears to have the potential to improve on the domain-general cognitive aspects shown to be affected by LI, and which underlie LI in theoretical constructs (see, e.g., Leonard et al., 2007, on limited processing capacity theory). If so, bilingual children with LI will present a unique linguistic and cognitive profile, distinct from those of both TD second language learners and monolinguals with LI (for a discussion, see Peets and Bialystok, 2010).

## Present Study

Below, we briefly outline the aims, method, and results of an on-going study investigating a possible bilingual advantage in the executive functions of Swedish–Arabic bilingual children with LI, followed by a discussion of the implications of the results for SLP services and research.

### Aims

To investigate whether bilingual Swedish–Arabic children with LI exhibit a bilingual advantage in executive functions.

### Method

Fifty-four children participated in assessment of short term memory [digit span forward, WISC-IV (Wechsler, 2004); verbatim number recall], working memory [digit span backward, WISC-IV (Wechsler, 2004), reverse order number recall], and inhibition [Berg Card Sorting Test (BCST; Mueller, 2010, sorting 128 cards according to undisclosed rules of number, color, and shape], to investigate executive functions as part of a larger study of bilingual lexical development. Prior to inclusion in the study, all participants with LI were diagnosed by a certified speech-language pathologist. Participants with TD were free from parental or teacher concern regarding language or attention. Initial analyses of receptive vocabulary, using conceptual scoring, taking into account knowledge in both languages of bilingual participants, showed equal performance between mono- and bilingual children, with and without LI, respectively (*p*’s > 0.4). LI and TD participants were recruited from the same schools in order to reduce possible differences in socio-economic factors. Recruitment of participants and assessments were approved by the Regional Ethics Review Board for southern Sweden, approval number 2010/717.

Socio-economic status was scored from the level of parental education; primary (compulsory schooling, 1), secondary (compulsory or non-compulsory, 2), or tertiary (university level, 3) education. Arabic was the first language of both parents to all bilingual participants, and Swedish the first language of both parents to all monolingual participants. All bilingual children attended Swedish-speaking schools and had attended Swedish preschools for more than 2 years prior to the assessment. Parental reports showed the participants to be exposed to Arabic primarily at home, and to Swedish in school. No bilingual participant was reported to use either language exclusively. All participants passed a 20 dB pure-tone hearing screening at 1, 2, and 4 kHz and performed above the 10th percentile on Raven’s Progressive Matrices. Mean values for participant characteristics and dependent variables are presented in **Table 1**.

**Table 1:** Mean values for participant characteristics and dependent variables.

	BLI (*n* = 9)	BTD (*n* = 18)	MLI (*n* = 9)	MTD (*n* = 18)	
	*M* (SD)	*M* (SD)	*M* (SD)	*M* (SD)	*p*
Age	6;10 (0;7)	7;0 (0;7)	6;10 (0;8)	6;11 (0;6)	0.75
SES	1.79 (0.49)	2.25 (0.59)	2.37 (0.52)	2.44 (0.63)	0.10
Arabic exposure (%)	53.0 (5.2)	50.9 (9.3)			0.59
Arabic use (%)	37.3 (15.3)	42.3 (14.6)			0.51
Digit span forward	-0.46 (0.58)	0.03 (1.0)	-1.06 (0.65)	0.73 (0.69)	<0.001
Digit span backward	-0.52 (0.89)	0.30 (0.56)	-0.97 (1.50)	0.45 (0.63)	<0.001
Berg Card Sorting Test (BCST)	-0.49 (1.17)	0.33 (0.92)	-0.82 (1.12)	0.32 (0.57)	0.004

*BLI, Bilingual LI; BTD, Bilingual TD; MLI, monolingual LI; MTD, monolingual TD*.

Assessments of digit span forward, digit span backward and BCST were performed in accordance with the procedures described in the WISC-IV (Wechsler, 2004) and BCST (Mueller, 2010) manuals. For the bilingual participants, assessment of digit span was conducted in both Arabic and Swedish. No significant difference in performance was found [forward: *t*(24) = 0.38, *p* = 0.70; backward: *t*(24) = 1.76, *p* = 0.10] and results for Swedish are used in all subsequent analyses and discussions.

## Results

**FIGURE 1:**
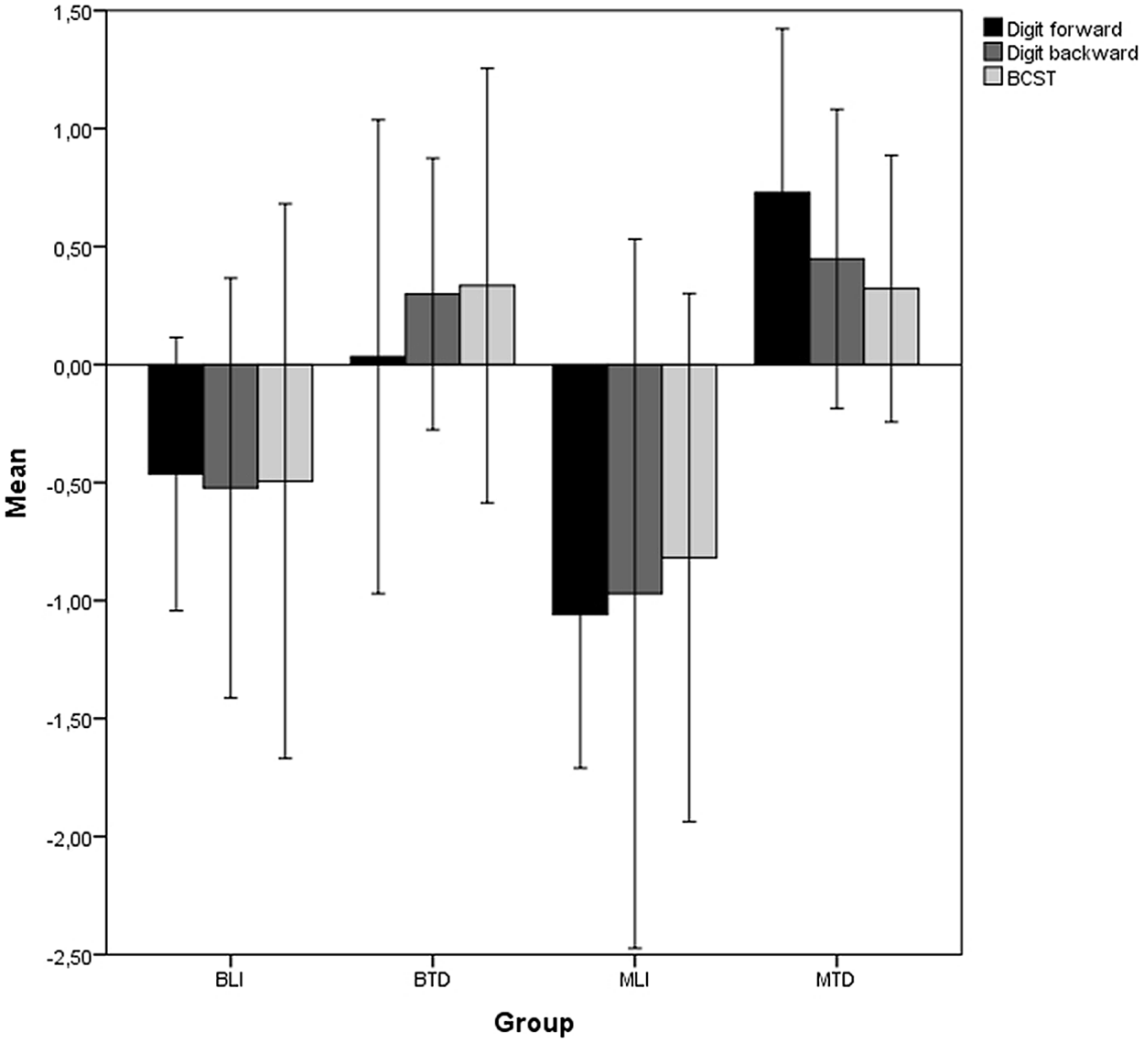
***Z*-scores with ±1 SD error bars for dependent variables (digit span forward, digit span backward, Berg Card Sorting Test).** BLI, Bilingual LI; BTD, Bilingual TD; MLI, monolingual LI; MTD, monolingual TD.

The results presented here are preliminary and should be interpreted accordingly. All raw scores were converted to *z*-scores. Correct responses on digit span forward, digit span backward and BCST were entered as dependent variables in a multivariate ANOVA with group as the independent variable. A statistically significant difference between the groups was found for an overall measure of executive functions, combining the scores of all dependent variables [*F*(9,150) = 4.12, *p* < 0.001, Pillai’s Trace = 0.60, $\eta_{p}^{2}$ = 0.20]. The group difference remained significant when the dependent variables were analyzed separately [digit span forward; *F*(3,50) = 11.46, *p* < 0.001, $\eta_{p}^{2}$ = 0.41, digit span backward; *F*(3,50) = 7.31, *p* < 0.001, $\eta_{p}^{2}$ = 0.31, BCST; *F*(3,50) = 4.93, *p* = 0.004, $\eta_{p}^{2}$ = 0.23; see **Figure 1** and **Table 1**]. *Post hoc* analyses with LSD revealed BLI to perform on par with MLI on all measures [digit span forward; *p* = 0.12, *d* = 0.96, digit span backward; *p* = 0.27, *d* = 0.36, BCST; *p* = 0.45, *d* = 0.28]. MTD outperformed BTD on digit span forward (*p* = 0.01, *d* = 0.81) while similar performance between TD groups was found for digit span backward (*p* = 0.60, *d* = 0.24) and BCST (*p* = 0.97, *d* = 0.01). For comparisons between LI and TD groups, BLI and BTD performed similarly on digit span forward (*p* = 0.13, *d* = 0.61) while BLI performed significantly below BTD on digit span backward (*p* = 0.02, *d* = 1.10) and BCST (*p* = 0.03, *d* = 0.78). MTD outperformed MLI on all measures [digit span forward; *p* < 0.001, *d* = 2.65, digit span backward; *p* < 0.001, *d* = 1.23, BCST; *p* = 0.003, *d* = 1.29].

To summarize, BLI and MLI performed on par on all dependent variables, while BTD and MTD differed only on digit span forward. BLI differed from BTD peers on digit span backward and BCST while MLI differed significantly from MTD on all measures. Digit span backward and digit span forward produced the largest effect sizes for BLI-BTD and MLI-MTD comparisons, respectively. For BLI-MLI and BTD-MTD comparisons, BCST produced the smallest effect sizes.

## Discussion

While preliminary, the results replicate earlier findings which indicate that measures of non-linguistic processing may provide important information in multilingual contexts (Paradis, 2010a,b). The study fails to provide evidence for a bilingual advantage in bilingual children with LI. Importantly, a bilingual disadvantage is also absent, somewhat surprisingly considering lower socio-economic status and lower Swedish language exposure for the bilingual than for the monolingual groups. The effects and interactions of socio-economic status (previously shown to attenuate a bilingual advantage in executive functions, see Morton and Harper, 2007), language proficiency (shown to affect cognitive processing in younger children, see Okanda et al., 2010), task complexity in relation to LI, and sample size may all play a role in explaining the absent bilingual advantage. While linguistic measures are commonly found to differ between mono- and bilingual children, equal performance in the present study indicates that executive functions are less subjected to influence from language exposure. Still, the measures appear to tap linguistic processing. For digit span forward, measuring short term memory, the best performance is found in monolinguals with TD, and the measure is also the best to separate monolinguals with and without LI. Interestingly, the bilinguals with and without LI show equal performance in digit span forward, a finding which could, as suggested by Morales et al. (2013), be interpreted as a bilingual advantage. The task of repeating digits may be complex enough to evoke an advantage for the bilinguals with LI, while their TD peers, with overall greater linguistic abilities, will not find the task challenging enough. In contrast, digit span backward, measuring working memory, appears to evoke an advantage also for bilinguals with TD, more clearly separating the bilingual children with and without LI for this measure.

The results of these preliminary analyses indicate that the clinical benefits of including executive functions in the assessment of LI are limited, at least in terms of identifying children with LI. Our sample is small, and replication is needed to see which results can be generalized. Subsequent studies should further investigate the influence of language proficiency on a bilingual advantage in executive functions. As suggested by Peets and Bialystok (2010), second language learners early in development may not show the effect, or show a bilingual advantage in other tasks than peers with more developed linguistic capacities. If LI is the result of atypical cognitive processes affecting, for example, executive functions, bilingualism might offset these processes, and improve language development. However, all children with LI may not exhibit deficits in executive functions, and further analyses must delve deeper into the interaction between executive functions and language ability, by investigating the individual language profile of participants with differences in executive functions. This may enable more individualized intervention, as well as improved differential diagnostics in speech-language pathology. For example, this may help determine the threshold in executive functions necessary for positive effects on language outcome, and contribute to a better understanding of the complex cognitive and language profiles of bilingual children with LI.

## Conflict of Interest Statement

The authors declare that the research was conducted in the absence of any commercial or financial relationships that could be construed as a potential conflict of interest.
